# Ti^3+^-self doped brookite TiO_2_ single-crystalline nanosheets with high solar absorption and excellent photocatalytic CO_2_ reduction

**DOI:** 10.1038/srep23684

**Published:** 2016-03-29

**Authors:** Xiaoye Xin, Tao Xu, Lan Wang, Chuanyi Wang

**Affiliations:** 1 Laboratory of Environmental Sciences and Technology, Xinjiang Technical Institute of Physics & Chemistry; Key Laboratory of Functional Materials and Devices for Special Environments, Chinese Academy of Sciences, Urumqi 830011, China; 2 Department of Chemistry and Biochemistry, Northern Illinois University, DeKalb, IL 60115, USA

## Abstract

Black brookite TiO_2_ single-crystalline nanosheets with outstanding photocatalytic activity toward CO_2_ reduction is prepared by a facile oxidation-based hydrothermal reaction method combined with post-annealing treatment. Large amount of Ti^3+^ defects are introduced into the bulk of brookite nanoparticles, which increases the solar energy absorption and enhances the photocatalytic activity.

The emission of greenhouse gases, particularly carbon dioxide (CO_2_), could result in the global climate change and unhealthful regional air quality^1^,^2^. To reduce the emission of CO_2_ and to achieve a sustainable energy future, novel materials and new promising “green chemistry” technologies are desired to convert CO_2_ into useful chemical compounds and fuels using sunlight. CO_2_ photoreduction in engineered systems, however, still faces severe challenges like low conversion yields and quantum efficiencies due to fast electron-hole recombination, narrow sunlight absorption range, and backward reactions^3^,^4^,^5^,^6^. Strategies to resolve those challenges have been a research hotspot in the area^7^,^8^,^9^,^10^. Among the studied semiconductor photocatalysts, titanium dioxide (TiO_2_) is by far the most popular catalyst because of its low cost, suitable bandgap and nature abundance^11^,^12^,^13^. From this prospect, TiO_2_ is still a promising photocatalyst for CO_2_ photoreduction due to its favorable band edges because TiO_2_ has a higher CB edge, which will promote the reaction of CO_2_ reduction with H_2_O that has a high reduction potential^14^.

TiO_2_ naturally exists in three polymorphs: anatase, brookite and rutile. Among them, only TiO_2_ anatase, rutile, and anatase-rutile mixed phase (e.g., Degussa P25) have been studied for CO_2_ photoreduction^15^. In other words, naturally occurring metastable brookite is the least investigated one because of the difficulties to obtain its pure form^16^. Based on the previous model for the structure-property relationship of photo-catalytic materials, the photocatalytic activity of brookite would be inferior to anatase but superior to rutile^17^,^18^,^19^. For brookite, lower packing factor, in accordance with higher structural openness degree, is associated with stronger ability for electron-hole separation and transfer, and usually results in better photocatalytic activity^20^,^21^. Considering that brookite has a relative open structure, its photocatalytic performance is worth investigating such as in CO_2_ photoreduction.

Many efforts have been made in decades to improve the photocatalytic activity of brookite, including preparation of nanosized brookite with various morphologies and studies of the involved shape-dependent photocatalytic performance^22^,^23^,^24^,^25^,^26^. However, the application of brookite TiO_2_ has been limited to the ultraviolet (UV) range, which makes it an inefficient photocatalyst for fully exploiting solar light. Narrowing down the TiO_2_ bandgap to turn it into an efficient material for solar energy conversion has been an ongoing challenge. Recently, the chemistry of structurally defective TiO_2_ with Ti^3+^ self-doping has been developed to solve the challenges in broad spectral range of photon absorption. Reduction of Ti^4+^ into Ti^3+^ is often achieved by harsh and costly physical methods, such as high temperature and pressurized hydrogenation^27^,^28^,^29^,^30^, plasma treatment^31^,^32^, vacuum activation and electron beams irradiation^33^ to improved light absorption and photocatalytic activities. Thus, a facile method is greatly desired to get defective brookite TiO_2_ with Ti^3+^ self-doping.

In this work, we developed an innovative facial oxidation-based hydrothermal method to synthesize bulk Ti^3+^ self-doped brookite TiO_2_ single-crystalline nanosheets (denoted as TiO_2−x_ if no further heat treatment). By post-annealing at different temperatures, Ti^3+^ self-doped brookite TiO_2_ with controllable amounts of Ti^3+^ defects is achieved. The black brookite TiO_2−x_ (T500) shows drastically enhanced visible-light absorption and exhibits excellent photocatalytic activity toward CO_2_ reduction.

## Results

**Figure 1:**
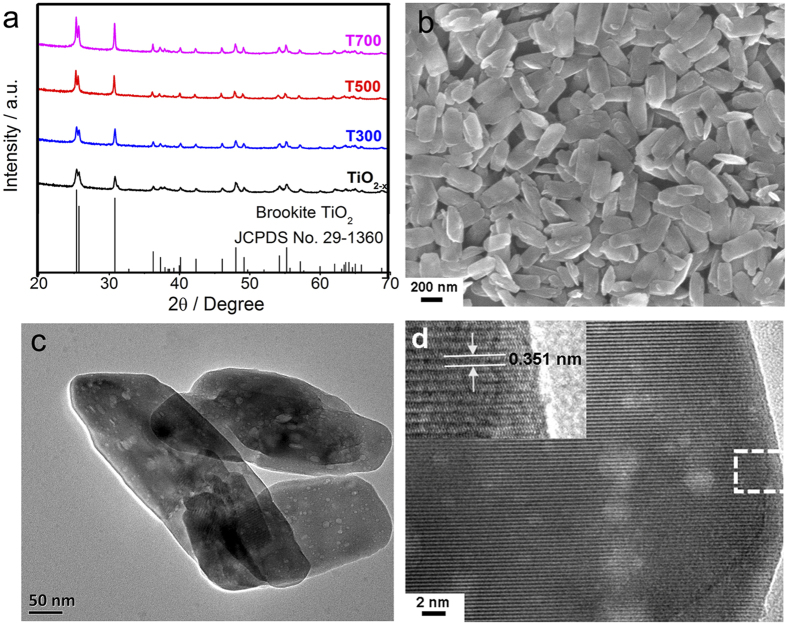
(**a**) XRD patterns of as-prepared brookite TiO_2−x_ and the blue TiO_2−x_ after postannealing treatment at different temperatures for 3 h. (**b**) SEM image of the typical T500 sample. (**c**) TEM image of the T500 sample. (**d**) HRTEM image taken from the edge of the single crystal.

The X-ray diffraction (XRD) patterns of the high quality brookite Ti^3+^-self doped TiO_2_ is displayed in Fig. 1a. The patterns show that all the samples are pure brookite phase without any other impurity phase (rutile or anatase phase). All the diffraction positions and intensity distributions can be indexed to the brookite corresponding to the JCPDS card No. 29–1360. By increasing the post-annealing temperature, the characteristic diffraction peaks of defective brookite TiO_2_ becomes stronger and sharper due to the increase in the crystallinity. When the temperature reaches 700 °C, no rutile or anatase characteristic diffraction peaks were detected, indicating that the brookite phase can be stabilized at higher temperatures.

The morphology of the synthesized brookite powders is investigated by scanning electron microscopy (SEM) and transmission electron microscopy (TEM) as shown in Fig. 1b,c, respectively. Clearly, the Ti^3+^-self doped brookite shows platelike features with uniform sizes of length up to 400 nm and thickness up to 10 nm. The obtained TiO_2_ particles are uniformly dispersed without obvious aggregation. There is no further particle growth with an increase of the post-annealing temperature. Distinct crystal planes and smooth fringes can be observed clearly from the magnified image of a single particle as shown in Fig. 1c.

**Figure 2:**
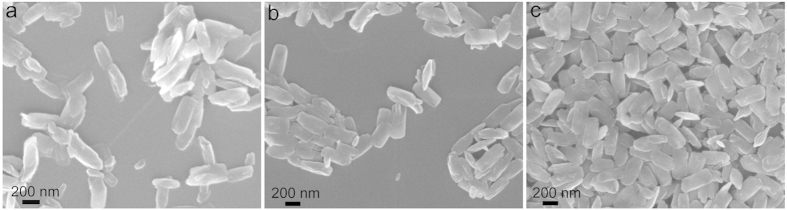
SEM images of (**a**) TiO_2−x_, (**b**) T300, and (**c**) T700.

In order to unveil the microstructure transformation of the brookite after post-annealing treatment, the TiO_2−x_ sample treated at 500 °C were examined by high resolution transmission electron microscopy (HRTEM) as shown in Fig. 1d. The uniform lattices indicate that the Ti^3+^ self-doped brookite TiO_2_ nanoparticles are highly crystallized. In addition, the spacing of the fringes paralleling to the top and bottom of the vertical nanoplate is 0.351 nm (Fig. 1d, inset), which can be attributed to the (210) facet of brookite. There is no obvious difference in lattice fringes width (e.g., those for rutile phase or TiH_2_) indicating that the as-prepared sample is pure brookite phase. This result is consistent with XRD analysis. Additionally, as shown in Fig. 2, there is no obvious difference among the other samples, indicating that the temperature change has very limited influence on the morphology and crystallite size of these nanocrystals.

**Figure 3:**
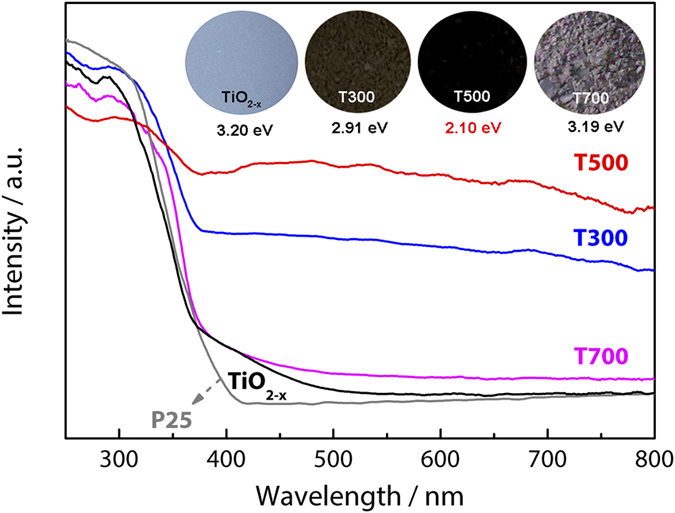
UV-vis diffuse reflectance spectra of the as-prepared brookite TiO_2−x_, T300, T500 and T700.

The top insert of Fig. 3 is photographic images of Ti^3+^ self-doped brookite TiO_2_ samples prepared with postannealing treatment at different temperatures for 3 h in a N_2_ gas flow and their optical band gap estimated by Kubelka-Munk function. The results show that the color of brookite TiO_2_ samples is remarkably different from white perfect TiO_2_ nanocrystal particles. From previous research, this phenomenon indicates the existence of Ti^3+^ defects^30^. The color of the brookite turns from blue to brown (T300) and further to black (T500). The color variation indicates the enhanced light absorption of brookite after post-annealing treatment up to 500 °C. As the post-annealing treatment temperature further increases, the dark black color is not maintained but gradually faded to a light grey blue at 700 °C.

Figure 3 displays the UV-vis diffuse reflectance spectra (UV-vis DRS) of as-prepared brookite TiO_2−x_, T300, T500 and T700 nanoparticles, and Deguass P25 as reference was also examined. The absorption peak at 390 nm is attributed to the intrinsic bandgap absorption of crystalline brookite TiO_2−x_. The as-prepared TiO_2−x_ sample (blue in color) exhibits an obvious stronger absorption between 400 and 800 nm compared to P25. This strong absorption is attributed to the existence of Ti^3+^ defects which induce a continuous vacancy band of electronic states just below the conduction band edge of TiO_2−x_. The existence of Ti^3+^ defects is supported by ESR measurements that will be discussed in later section. Compared with TiO_2−x_, the absorption in the visible light region is greatly enhanced after post-annealing treatment. As the annealing temperature increases from 300 °C to 500 °C, the light absorption increases gradually, in good agreement with the color transformation of brookite. However, the light absorption of T700 presents a dramatically decrease in comparison with the T500 sample, which is coincident with the observed color change trend of the samples (as depicted in the insert of Fig. 3). In the hydrothermally treated brookiteTiO_2−x_, the enhanced visible-light absorption is attributed to the fact that hydrothermally treated process introduces the disorder in the TiO_2_ and the resulted bandgap narrowing^34^, For the black brookite TiO_2_, postannealing treatment not only increases the structural openness, but also introduces Ti^3+^ (3d^1^) in the TiO_2−x_ bulk^35^, Using the Kubelka–Munk function as the vertical axis to plot it against the photon energy, the optical band gaps of all samples can be derived, and the results are summarized in the top insert of Fig. 3. Among all the samples, the band gap of T500 (2.10 eV) is the narrowest one. These aspects may work together leading to the black colouration, enhanced light absorption and promoted photocatalytic activity of defective brookite TiO_2_.

**Figure 4:**
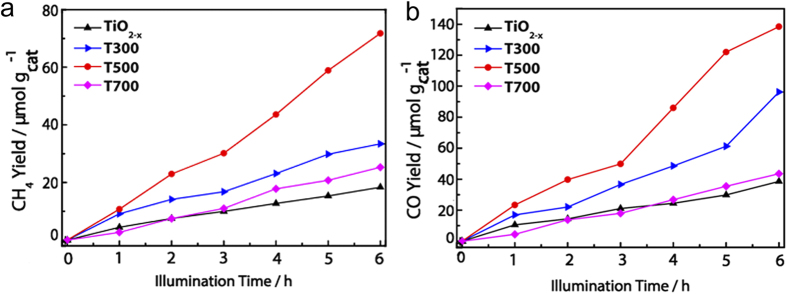
(**a**) CH_4_ and (**b**) CO evolution over the brookite TiO_2−x_, T300, T500 and T700 samples for a period of 6 h visible-light illumination (λ ≥ 420 nm).

In this work, photoactivity of as prepared defective brookite TiO_2−x_ was explored by the heterogeneous photoreduction of CO_2_ under visible light illumination (Fig. 4). CH_4_ and CO were detected as the major hydrocarbon products from the photoreduction of CO_2_ over the as-synthesized Ti^3+^-self doped brookite TiO_2_ photocatalysts. The yields of CH_4_ and CO (μmol/g_cat_) attained after 6 h of visible-light irradiation are shown in Fig. 4a,b (detailed calculation can refer to Eq. 1).


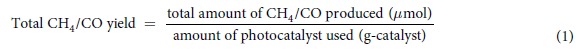


## Discussion

**Figure 5:**
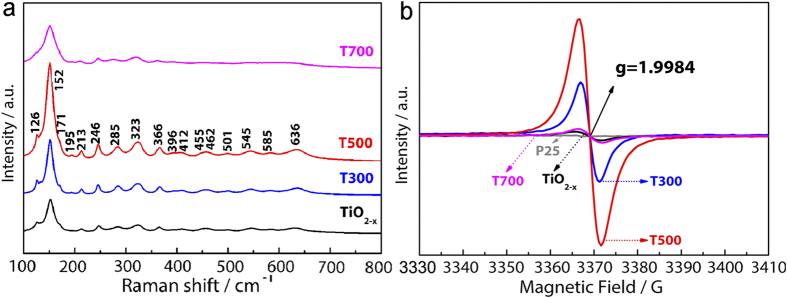
(**a**) Raman spectra and (**b**) low-temperature EPR spectra of the brookite TiO_2−x_, T300, T500 and T700 samples.

The low symmetry and large unit cell of brookite result in a diagnostically complex Raman spectrum compared to the other two polymorphs (anatase and rutile), thus Raman spectroscopy has been widely used to detect the presence of the brookite phase in TiO_2_ containing materials^25^. Therefore, Raman spectra of the before and after post-annealing treatment can further confirm the phase composition. As shown in Fig. 5a, the TiO_2−x_ and the annealed defective TiO_2_ at different temperatures display the typical Raman spectra of brookite, and 16 bands are identified in total, including seven A_1g_ (124, 151, 192, 244, 410, 544, 636 cm^−1^), four B_1g_ (211, 318, 415, 500 cm^−1^), four B_2g_ (365, 393, 460, 581 cm^−1^), and one B_3g_ (287 cm^−1^). The characteristic Raman signals at 399 cm^−1^ and 519 cm^−1^ for anatase or at 447 cm^−1^ and 612 cm^−1^ for rutile are not observed. This further proves the high phase purity of brookite for the TiO_2−x_ and the annealed defective TiO_2_ samples, in good agreement with the XRD results.

In addition, the intensity of high-resolution Eg Raman vibration modes increases as the post-annealing treatment temperature increases up to 500 °C. This is attributed to the effect of enhanced crystallinity upon the increase in annealing temperature, because higher crystallinity contains more Raman active facets, which is proportional to the Eg intensity fluctuations. When the temperature increases to 700 °C, the intensity of Raman vibration modes is decreased and the peak width is broadened, which may be due to the change in the distribution and concentration of Ti^3+^. The fact that the Ti^3+^-doped TiO_2_ has quite different high-resolution Eg modes in Raman spectra with different defects distribution and concentration suggests that Ti^3+^ should mainly localize within the bulk of the samples.

Electron paramagnetic resonance (EPR), which is highly sensitive to paramagnetic species containing unpaired electrons, has been widely used to characterize the existence of Ti^3+^ defects. As indicated in Fig. 5b, the pristine TiO_2_ contains almost completely Ti^4+^ (3d^0^) and shows a negligible paramagnetic signal peak under the present EPR measurement condition at 110 K. In general, the surface Ti^3+^ tends to adsorb O_2_, which could be reduced to superoxide radical anions (O^2−^) with an EPR signal feature at g = 2.02^36^. However, no such signal appears for all samples, further demonstrating the absence of surface Ti^3+^.

On the other hand, the EPR spectra of brookite defective TiO_2_ nanoparticles show intense axially symmetry signals centered at the g value of 1.9984, which have been reported for Ti^3+^ ions in brookite crystallite^37^, indicating that the free electrons occupy interior Ti position thereby generating Ti^3+^ defects in bulk. Accordingly, the chemical equation in our work can be expressed as below:


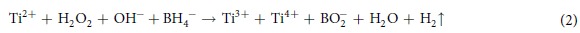


Furthermore, the EPR intensity increases along with the post-annealing temperature. The strongest value is obtained for T500, but the signal intensity declines sharply when further increasing the treatment temperature to 700 °C. By numerical double integration of the EPR spectra with an aqueous solution of Cu^2+^ as reference, the amount of Ti^3+^ centers for TiO_2−x_ sample is calculated to be 0.7 × 10^19 ^spins/mol, equivalent to one Ti^3+^ out of every 2.9 × 10^4^ Ti^4+^. Whereas, the amount of Ti^3+^ centers for T500 sample is almost 10 times larger than that for TiO_2−x_, namely, one Ti^3+^ in every 2.9 × 10^3^ Ti^4+^. This suggests that the concentration of bulk Ti^3+^ defect is greatly enhanced by the high temperature post-annealing treatment. From our previous study, the decreased Ti^3+^ defects upon treatment at 700 °C is mainly due to the difficulty of further diffusion of Ti^3+^ in enhanced crystallinity, leading to the dilution of the “colour center”^38^.

**Figure 6:**
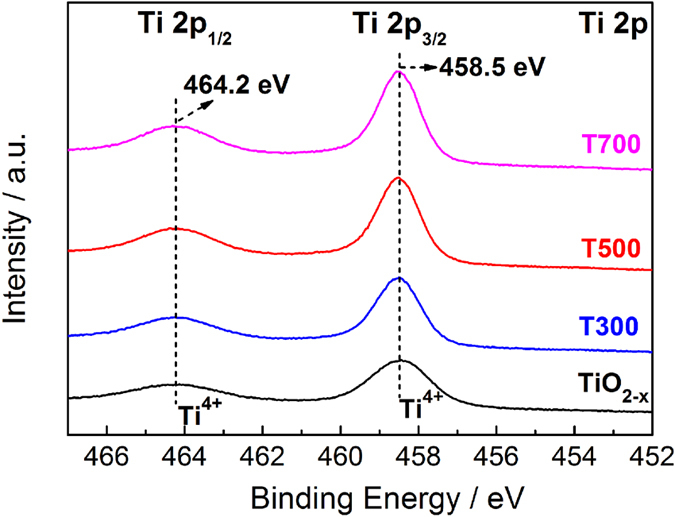
Ti 2p XPS spectra of the brookite TiO_2−x_, T300, T500 and T700 samples.

In order to confirm the existence of Ti^3+^, additional characterizations are performed. X-ray photoelectron spectroscopy (XPS) is performed to further investigate the transformation of surface chemical bonding and to detect the electronic valence band position of the brookite samples. As shown in Fig. 6, the Ti 2p XPS spectra of brookite TiO_2−x_, T300, T500 and T700 samples show the typical pattern of Ti^4+^ –O bonds in TiO_2_ with Ti 2p_3/2_ and 2p_1/2_ peaks centered at binding energies of 458.5 eV and 464.2 eV^39^. For all the samples, no peaks shift apparently to lower energy, indicating that Ti^3+^ species are separately located at bulk of the samples, and no different chemical states and disorders on the surface of samples. This agrees with the HRTEM observation, i.e., no obvious disordered layer in the edge of the samples.

**Figure 7:**
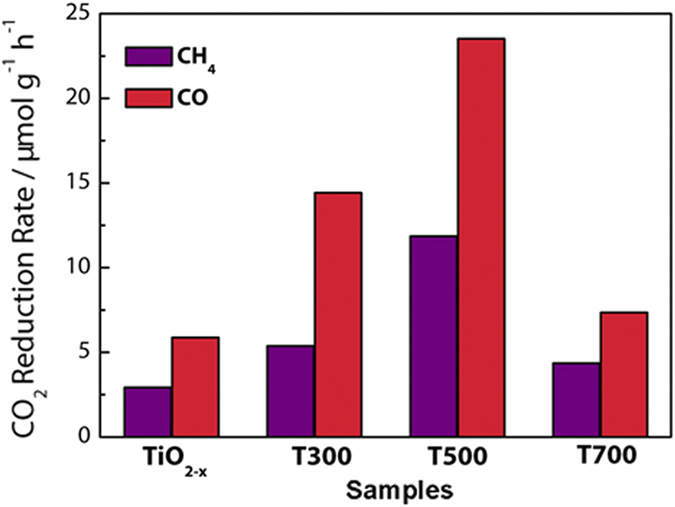
CO_2_ reduction rate of the brookite TiO_2−x_, T300, T500 and T700 samples.

The CO_2_ reduction rate is presented in Fig. 7. The CO_2_ reduction rate of annealed brookite TiO_2_ also improves dramatically; it shows drastically enhanced CO_2_ reduction rate when brookite TiO_2−x_ is annealled at 500 °C (11.9 μmol·g_cat_^−1^h^−1^ for CH_4_ and 23.5 μmol·g_cat_^−1^h^−1^ for CO). However, defective brookite prepared at 700 °C shows lower CO_2_ reduction rate (4.4 μmol·g_cat_^−1^h^−1^ for CH_4_ and 7.3 μmol·g_cat_^−1^h^−1^ for CO), but is still higher than untreated TiO_2−x_ sample. It can be deduced that the photocatalytic reduction performance agrees well with the light absorption and Ti^3+^ defects (numbers and distribution); the more the light absorption and the Ti^3+^ defects, the higher the photocatalytic reduction activity is.

Clearly, the engineered Ti^3+^-self doped brookite catalysts developed in this work demonstrate a superior activity. It is the first time to present a facile approach to controllablly synthesize Ti^3+^-self doped brookite TiO_2_ as an outstanding candidate for CO_2_ photoreduction to produce CO and CH_4_. Further study on the selectivity of CO_2_ reduction is going on.

In summary, this work illustrates that the oxidation-based hydrothermal synthesis of Ti^3+^ self-doped brookite TiO_2_ is an effective strategy to prepare uniform Ti^3+^ self-doped brookite TiO_2_ nanosheets. The introduction of Ti^3+^ defects in the bulk of brookite enhances the visible light absorption and narrows the bandgap. Our study demonstrates that brookite can be successfully tuned to be highly active toward photocatalytic performance for CO_2_ reduction through post-annealing at different temperatures, which illuminates the future research of brookite; the oxidation-based hydrothermal synthesis combined with post-annealing treatment may provide a novel path for tuning the photocatalysts from relatively inert to highly active and shine light on the greenhouse gases conversion into sustainable energy.

## Methods

### Materials

Titanium hydride (TiH_2_, 98.0%) powder was purchased from Sigma–Aldrich Co., LLC. Hydrogen peroxide (H_2_O_2_, 30.0%), sodium borohydride (NaBH_4_, 98.0%), hydrochloric acid (HCl, 36–38%, A.R.) and sodium hydroxide (NaOH, 98%, A.R.) were obtained from Sinopharm Chemical Reagent Co., Ltd. and used as received without any further purification. Double distilled water was used throughout the experiments.

### Synthesis of Ti^3+^ self-doped brookite TiO_2_ nanoparticles

In a typical synthetic process, TiH_2_ (0.256 g) and H_2_O (2 mL) were mixed in a 50 mL round-bottomed flask magnetically stirred for 5 min. Then H_2_O_2_ (30 mL 30.0 wt%) as the oxidation was added dropwise to this dark gray suspensions, and this mixture was vigorously stirred for 12 h till it changed to yellowish gel-like state. After that, double distilled water (40 mL) was added under continuous magnetic stirring. A certain amount of NaOH (1.0 M) solution as the pH regulator was added gradually until the pH of the mixture solution was tuned to 9.0. NaBH_4_ (0.4 g) as reducing agent was added to this light yellow transparency mixture and then transferred to the Teflon-lined stainless-steel autoclave immediately and hydrothermally treated at 180 °C for 24 h. The sample was then collected and added into HCl (50.0 mL, 1.0 M) solution to eliminate the sodium boron compounds. After stirring for 10 h, the powders were washed by distilled water and ethanol repetitively to remove the impurities (e.g., Na^+^, Cl^−,^ BO_3_^2−^). The obtained precipitate was dried under vacuum for 12 h to yield a grey blue TiO_2_ nanocrystals powder, denoted as TiO_2−x_. Postannealing treatment of the TiO_2−x_ sample was conducted under a N_2_ gas flow (150 sccm) in a tube furnace at an elevated temperature of 300 °C, 500 °C and 700 °C for 3 h. Therefore, the samples obtained at the specific temperatures are designated as T300, T500 and T700, respectively.

### Characterization

The crystal structures of the samples were identified on a Bruker D8 X-ray diffractometer with Cu Kα radiation (λ = 0.15418 nm). The morphology photographs of the samples were recorded by field emission scanning electron microscopy (FESEM; ZEISS SUPRA55VP) and transmission electron microscopy (TEM, JEOL-JEM 2100). Ultraviolet–visible (UV–vis) diffusion reflectance spectra of the samples were obtained on a SolidSpec-3700DUV spectrophotometer (Shimadzu) using BaSO_4_ as reference to obtain absorption spectra for determining the band gap. Raman spectra were obtained on a laser Raman spectrometer (LabRAM HR Evolution RAMAN SPECTROMETER, HORIBA Scientific Ltd.) with a back scattering configuration using an Ar^+^ laser (20 mW, 532 nm) as excitation source. The surface electronic state analysis was studied by X-ray photoelectron spectra (XPS), and the measurements were carried out on an X-ray photoelectron spectrometer (ESCALAB MK II) using Mg Ka (1253.6 eV) X-rays as the excitation source, with C 1s (284.6 eV) for calibration. Electron paramagnetic resonance (EPR) spectra were recorded on a Bruker Elexsys E500 spectrometer by applying an X-band (9.43 GHz, 1.5 mW) microwave with sweeping magnetic field at 110 K in cells that can be connected to a conventional high-vacuum apparatus (residual pressure <10^−4 ^mbar). The concentration of Ti^3+^ was determined by a numerical double integration of the EPR spectra in comparison with an aqueous solution of Cu^2+^.

### Photocatalitic reduction of CO_2_ under visible-light

The photocatalytic activities of the Ti^3+^-self doped brookite TiO_2_ single-crystalline nanosheets were studied using a CO_2_ photoreduction system at ambient condition in a continuous gas flow reactor. The CO_2_ photoreduction process was performed under visible light irradiation with a 300 W Xe lamp (PLS-SXE300, Perfect Light Company, Beijing, China) equipped with an ultraviolet cut-off filter to provide visible light (≥420 nm). The illumination intensity at the surface was 0.216 W/cm^2^ as measured by a calibrated precision optical power meter (1916-C, Newport Corp.). The amount of photocatalyst used was held constant in all runs. Highly pure CO_2_ (99.99%) was bubbled through water to produce a mixture of CO_2_ and water vapor into the photoreactor at atmospheric pressure. Before switching on the light source, wet CO_2_ was permitted to flow through the photoreactor at 30 mL/min for 30 min to eliminate any excess air and to ensure the complete adsorption of gas molecules. The photoreactor was operated in a continuous flow mode (2.0 mL/min flow rate), and the gaseous products in the reactor effluent were continuously analyzed for 6 h by a gas chromatograph (GC, Agilent 7890 A) equipped with both a thermal conductivity detector (TCD) and a flame ionization detector (FID).

## Additional Information

**How to cite this article**: Xin, X. *et al*. Ti^3+^-self doped brookite TiO_2_ single-crystalline nanosheets with high solar absorption and excellent photocatalytic CO_2_ reduction. *Sci. Rep*. **6**, 23684; doi: 10.1038/srep23684 (2016).
